# An educational intervention to reduce the use of potentially inappropriate medications among older adults (EMPOWER study): protocol for a cluster randomized trial

**DOI:** 10.1186/1745-6215-14-80

**Published:** 2013-03-20

**Authors:** Philippe Martin, Robyn Tamblyn, Sara Ahmed, Cara Tannenbaum

**Affiliations:** 1 InstitutUniversitaire de Gériatrie de Montréal, Faculté de Pharmacie, Université de Montréal, Montréal, QC, Canada; 2 Department of Epidemiology, Biostatistics and Occupational Health, McGill University, Montréal, QC, Canada; 3 Faculty of Medicine, School of Physical & Occupational Therapy, McGill University, Montréal, QC, Canada

**Keywords:** Patient education, Benzodiazepine use, Inappropriate prescription, Older adult health, Cognition disorders, Drug therapy, Polypharmacy

## Abstract

**Background:**

Currently, far too many older adults consume inappropriate prescriptions, which increase the risk of adverse drug reactions and unnecessary hospitalizations. A health education program directly informing patients of prescription risks may promote inappropriate prescription discontinuation in chronic benzodiazepine users.

**Methods/Design:**

This is a cluster randomized controlled trial using a two-arm parallel-design. A total of 250 older chronic benzodiazepine users recruited from community pharmacies in the greater Montreal area will be studied with informed consent. A participating pharmacy with recruited participants represents a cluster, the unit of randomization. For every four pharmacies recruited, a simple 2:2 randomization is used to allocate clusters into intervention and control arms. Participants will be followed for 1 year. Within the intervention clusters, participants will receive a novel educational intervention detailing risks and safe alternatives to their current potentially inappropriate medication, while the control group will be wait-listed for the intervention for 6 months and receive usual care during that time period. The primary outcome is the rate of change in benzodiazepine use at 6 months. Secondary outcomes are changes in risk perception, self-efficacy for discontinuing benzodiazepines, and activation of patients initiating discussions with their physician or pharmacist about safer prescribing practices. An intention-to-treat analysis will be followed.

The rate of change of benzodiazepine use will be compared between intervention and control groups at the individual level at the 6-month follow-up. Risk differences between the control and experimental groups will be calculated, and the robust variance estimator will be used to estimate the associated 95% confidence interval (CI). As a sensitivity analysis (and/or if any confounders are unbalanced between the groups), we will estimate the risk difference for the intervention via a marginal model estimated via generalized estimating equations with an exchangeable correlation structure.

**Discussion:**

Targeting consumers directly as catalysts for engaging physicians and pharmacists in collaborative discontinuation of benzodiazepine drugs is a novel approach to reduce inappropriate prescriptions. By directly empowering chronic users with knowledge about risks, we hope to imitate the success of individually targeted anti-smoking campaigns.

**Trial registration:**

ClinicalTrials.gov identifier: NCT01148186

## Background

Appropriate and safe prescribing for older adults is rendered difficult by the increased risk of side effects, drug-drug interactions and adverse events, due to associated comorbidities and high prevalence polypharmacy in this population [1,2]. Prescriptions are considered inappropriate when potential risks outweigh potential benefits, and safer therapeutic alternatives exist that have similar or superior efficacy [3-5]. Avoiding the use of inappropriate and high-risk drugs is an important, simple and effective strategy in reducing medication-related problems and adverse drug events in older adults [5]. The Beers Criteria for Potentially Inappropriate Medication Use in Older Adults identifies, grades and qualifies potentially inappropriate medications. The criteria were developed by a panel of geriatric pharmacy experts who applied a modified Delphi method to a systematic review of all medications and graded the evidence to reach a consensus on a recommended list of drugs to avoid in older people [5-7].

Currently, far too many older adults are taking inappropriate prescriptions, which further increases the risk of adverse drug reactions and unnecessary hospitalizations [5,8-11]. Inappropriate prescribing has been estimated to occur in 12 to 40% of community-dwelling non-hospitalized older adults aged over 60 years, depending on the criteria used and the country studied [3,5,9-14]. A conservative estimate of the incremental healthcare expenditures related to inappropriate prescribing among community-dwelling older adults is $7.2 billion in the United States [12].

Benzodiazepines represent one of the most prevalent inappropriate prescriptions, consumed by 19% of older adults (range 10 to 42%) [15]. The new Beers list, released in 2012, recommends that all short- and long-acting benzodiazepine sedative-hypnotic drugs used for the treatment of anxiety and insomnia should be avoided in older adults, due to an excessive risk of delirium, falls, fractures and motor vehicle accidents [5,16-19]. Benzodiazepines have also been shown to increase the risk of amnestic and non-amnestic cognitive impairment and may lead to incident dementia [20,21].

Previous research has attempted to define the best strategy to inform and educate relevant parties, to try and implement safer prescribing practices, and to eliminate benzodiazepine use. The problem is that chronic benzodiazepine users develop a psychological dependence to benzodiazepines, and both physicians and consumers have difficulty implementing tapering protocols [22]. Many patients deny or minimize side effects, or express reluctance to risk suffering without these medications [22]. For these reasons physicians are hesitant about insisting on benzodiazepine discontinuation for fear of upsetting the doctor-patient relationship or because they believe that the patient tolerates the medication with minimal side effects [23].

Interventions to reduce benzodiazepine use in older people have been tested [24-47]. Several approaches have yielded insignificant results; other approaches, such as physician-targeted online drug audits, didactic educational activities and letters from physicians advising on risks associated with benzodiazepine use, have resulted in discontinuation rates ranging from 16 to 25% [43-47]. Despite achieving mild success in benzodiazepine discontinuation, these approaches are rarely feasible on a large scale and can be linked to extensive fees.

Targeting consumers directly as catalysts for engaging physicians and pharmacists in collaborative discontinuation of benzodiazepine drugs is a novel approach to reduce inappropriate prescriptions that has never been tested. Studies have shown that collaborative efforts to taper benzodiazepine use do not result in an increased workload for family physicians [48]. This type of approach could empower patients to participate in medication safety, diminish physician workload and do so at lower costs than current approaches in changing medical practice.

The aim of the current cluster randomized controlled trial is to determine the effectiveness of an educational tool directed at older adults on subsequent cessation of benzodiazepine use.

## Methods/Design

### Trial design

#### Study objectives

The primary objective of the EMPOWER trial is to evaluate the effectiveness of a new knowledge transfer tool on a community-based sample of chronic benzodiazepine users, as measured by the rate of benzodiazepine discontinuation at 6 months with 1-year follow-up, to determine whether change rates are sustained over the long-term. The acronym EMPOWER stands for “Eliminating Medications through Patient OWnership of End Results”.

Secondary objectives are to determine whether receipt of a knowledge transfer tool by chronic benzodiazepine users changes risk perceptions and self-efficacy for discontinuing benzodiazepines, and leads patients to initiate discussions about safer prescribing practices with their physician or pharmacist.

#### Design

**Figure 1:**
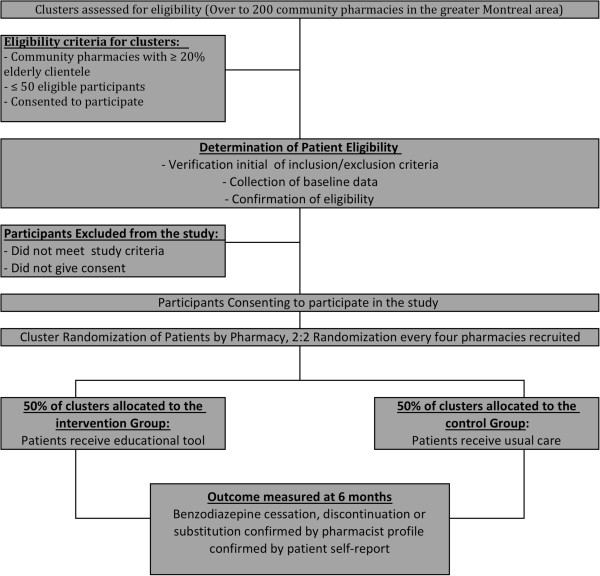
Study flow chart.

This is a cluster randomized controlled trial. The rationale for choosing a cluster design is to prevent contamination across the intervention and control arms by individual clients served by the same pharmacy. The cluster and unit of randomization is the community pharmacy. There are two arms in this parallel randomized controlled trial: the educational intervention arm and the control arm. A 50:50 ratio of participants will be used in each study arm. Figure 1 illustrates the study flow.

#### Study site: clusters and characteristics

The study is being conducted in the greater Montreal area in Quebec, Canada. Collaboration with a drugstore chain was established, and all pharmacies within a 3-hour driving radius (approximately 200 km) of Montreal were identified and listed. Pharmacies were listed in random order by a computer generated program, contacted sequentially and screened for eligibility criteria. Clusters consist of community pharmacies with ≥20% older adults. In order to prevent small or empty clusters, pharmacies with ≤50 eligible participants following the initial screening process are not recruited to the trial.

### Study population

The study population comprises chronic benzodiazepine users aged 65 years and older.

#### Eligibility criteria for individual patients to enroll in the study

Selection of participants will be according to the following inclusion and exclusion criteria.

#### Inclusion criteria

1. Men and women aged 65 years and older.

2. With at least five active prescriptions (polypharmacy).

3. Of which one is an active benzodiazepine prescription that has been dispensed for at least 3 consecutive months prior to screening, based on pharmacy records.

4. Patients who are willing to participate in the study.

#### Exclusion criteria

1. A diagnosis of severe mental illness or dementia ascertained by the presence of an active prescription for any antipsychotic medication, and/or a cholinesterase inhibitor or memantine in the preceding 3 months.

2. Unable to communicate in French and/or English.

3. Evidence of significant cognitive impairment (score under 21 on the Montreal Cognitive Assessment (MoCA) [49]).

4. Patients living in a long-term care facility.

#### Ethical approval

The study protocol was approved on 26 July 2009 by the Research Ethics Board of the Centre de Recherche de l’Institut Universitaire de Gériatrie de Montreal, Canada (ClinicalTrials.gov identifier: http://NCT01148186).

#### Enrollment

Enrollment in the trial is conducted in collaboration with a regional pharmacy chain. A letter from the vice-president of the chain was sent to all affiliated pharmacies inviting pharmacists to participate in the trial by recruiting eligible clients served by their medication dispensing units. Company headquarters then identified a list of all chain drugstores within a 3-hour driving radius of the research center and sent a list to the research team. This list was sorted in random order by a computer generated program and pharmacies are contacted systematically to ascertain their interest in participating in the study. Pharmacies interested in participating are supplied with a list of eligible participants identified from the company’s centralized electronic database by a preset inclusion/exclusion filter that applies all inclusion and most exclusion criteria. Any pharmacy found to have less than 50 potential candidates is excluded from the project to avoid small or empty clusters. Otherwise, pharmacies are enrolled in the study and proceed with participant recruitment.

#### Recruitment of participants and application of eligibility criteria

Recruitment of participants occurs through a three-step screening process. First, pharmacy clients are filtered by the company’s centralized computer system using preset eligibility criteria for age and medication use. Second, participating pharmacists receive a list of eligible clients with a matching set of personalized name and address labels from company headquarters through internal mail, and are asked to review the list to exclude patients with undetected dementia or those living in care facilities. Using the final list of potential participants, pharmacists tally the numbers and contact the research team to request an appropriate number of English and French study invitational materials intended for mailed distribution to participants.

Invitational materials consist of a headquarters pre-approved invitation letter personalized on behalf of the pharmacist and an accompanying brochure describing a study on ‘better drug management’. The flyer invites participants to contact either their pharmacist directly or the study coordinator by phone if they have any questions or are interested in participating in the study. Letters and invitations are put in envelopes by the pharmacy personnel, affixed with the address labels provided by company headquarters and mailed to all eligible participants.

One week after sending out the invitations, the pharmacist notes all replies spontaneously received from potential participants indicating their willingness or refusal to participate in the study. The pharmacist then calls the remaining candidates to ascertain their interest in participating in the study and, if so, to obtain permission to give their names and phone numbers to the study coordinator. According to protocol, a maximum of three phone calls and voice messages must be attempted over a 2-week time period in order to reach participants, after which time potential participants are declared not interested. All affirmative responses are recorded by the pharmacist, and the names and phone numbers of interested clients are transferred to the research staff at the end of the 3-week period following the invitation mail-out to participants.

The study coordinator then contacts all potential participants referred by the pharmacists (with the client’s permission) and arranges an appointment at the person’s residence to complete the third screening stage: signed consent if eligible and collection of baseline data. During the home visit, a research assistant reviews the medication currently taken by the patient, queries the medical history and administers the MoCA. Signed consent to participate in the study is then obtained from individuals who meet the study criteria after baseline cognitive and health status screening. All baseline data are collected from the questionnaires indicated in Table 1 under T0 at this time.

**Table 1:** Overview of data collection and measurements in both trial arms

	**Baseline**	**Follow-up post-intervention**			
Visit number	T0	T1	T2	T3	T4
Time	**1-7 months pre-intervention**	7 days	6 weeks	6 months	1 year
Inclusion and exclusion criteria	X				
Socio-demographic characteristics	X				
SMAF questionnaire	X				
GHS questionnaire	X				
MoCA	X				
Rey 15-Item Memory Test	X				
GAI	X		X	X	X
Depression PHQ-9	X^a^		X	X	X
Insomnia questionnaire	X^a^		X	X	X
Medication use characteristics	X				
Benzodiazepine tapering questionnaire		X	X	X	X
Medication knowledge questionnaire	X	X			
BMQ-Specific	X	X			
Self-efficacy scale	X	X			
Intervention related questionnaire		X	X	X	
Intervention appreciation questionnaire				X	

^a^Only administered if related outcome present. BMQ-Specific, Beliefs about Medicines Questionnaire - Specific segment; GAI, Geriatric Anxiety Inventory; GHS, general health status; MoCA, Montreal Cognitive Assessment; PHQ, Patient Health Questionnaire; SMAF, functional autonomy measurement system.

### Randomization

#### Randomization

A statistician, blinded to pharmacy and cluster size, generates a random allocation sequence using computer generated random digit numbers. For every four pharmacies recruited, a simple 2:2 randomization is used to allocate the four clusters into intervention and control groups. Towards the end of recruitment, randomization might be skewed to favor the least populated study arm to allow the desired 50:50 allocation ratio.

#### Concealment of allocation

Prior to random allocation into either arm of the study, informed consent, agreement to enroll in the study and ascertainment of eligibility will all be obtained from the pharmacists and their clients. Up until the point of randomization, neither the research assistant, the cluster representative (the pharmacist), nor the client will know the allocation of the clusters. After randomization, only the research assistant will be aware of treatment allocation. Pharmacists and participants will not be informed, and will remain unaware of the fact that there is another group in the study; nor will they be informed of the procedures for the other arm. Participants’ link to the project will be the pharmacist, but participants of the same pharmacy will not normally be in contact with each other. Randomization is performed in clusters to prevent bias in case this happens. Therefore all participants from the same pharmacy will be randomized as a single cluster, thereby receiving the same treatment and remaining blinded to treatment allocation.

#### Blinding

As the intervention is educational in nature, blinding of the intervention is impossible. However, to preserve a certain level of blinding and to protect sources of bias, the following measures are taken.

For participants, blinding is achieved by presenting the project to participants as a project on optimizing medication management. Consenting participants understand that their medication profiles will be transmitted to the research team within the following months and that they will receive a customized letter at some point during the year which may contain recommendations for change, which they can then decide to take to their physician or pharmacist for discussion.

For pharmacists, blinding is achieved by presenting the same study timeline. Pharmacists are aware that their clients will receive an intervention at some point during the following year and remain blinded to group allocation throughout the course of the study. Pharmacists also remain blinded to other participating pharmacies. Since pharmacies are randomized as clusters, they are located in distinct geographic locations and generally have no reason to interact with one another.

Thus, blinding pertains to both the individual and cluster level.

### The educational intervention

The educational intervention consists of a seven-page letter-size paper brochure developed specifically for this trial. The language for the intervention is set at a grade six reading level and written in 14 point font to facilitate accessibility of the material. The brochure is mailed to the intervention group within 1 week of group allocation. The control/wait-list group receives the educational tool 6 months later. As the intervention is sent individually to participants and participants within each cluster are unknown to one another, the intervention only pertains to the individual participant.

#### Theory and development of the intervention

The tool aims to promote active learning by using constructivist learning theory principles, incorporated during the development of the intervention. Constructivist learning theory activates users to create new knowledge in order to make sense out of the presented material. The goal of this approach is to allow the learner to interact with the academic material, fostering their own selecting, organizing and information integrating processes [50]. Many other learning theories were integrated in the different parts of the intervention, such as cognitive dissonance, social comparison, peer champion theories and self-assessment theory. Cognitive dissonance theory confronts two inconsistent cognitions held simultaneously by the same individual. This process aims to create an aversive motivational state in the individual who will then seek to alter one of these perceptions to remove the pressure caused by this conflict [51]. The tool also includes elements of social comparison and peer champion theories [52]. Social comparison consists of comparing oneself to others in order to evaluate or enhance personal aspects [53]. Thus, the evaluation of the ability or inability to accomplish a certain action depends on a proxy performer’s success. The efficacy of social comparison depends on whether the comparer assimilates or contrasts him/herself to others [52]. Thus, aspects such as previous agreement with the peer’s views and comparability with the peer champion are paramount for the comparison to work [53]. A self-assessment component was also introduced to promote insight about potential misinformation or beliefs held about benzodiazepine use [54,55]. A common idea in models of risk perception is that risk is perceived from two dimensions: knowledge of and beliefs. Information about the risks associated with benzodiazepine use was therefore in incorporated into the tool. It has also been shown that pre-existing beliefs frequently supersede information transfer about risks [56]. In order to understand the drivers and consequences of risk perception the behavior motivation hypothesis was used. This hypothesis, which is endorsed by most models of health behavior, describes the determinants of risk perception and their effects on behavior change [57]. It is important to note that perception of risk has been shown to be positively related to preventive health behavior in conditions where expectations of success in dealing with the risk are acceptable and when recommendations for preventive behavior are presented as effective [58].

The textual content of the intervention was based on guidelines concerning the use of benzodiazepines in older people as well as a systematic review of the evidence. The initial content of the tool was drafted by a geriatrician and graduate student, and then validated by a panel of colleagues with expertise in geriatric pharmacy. Following validation, a health librarian reviewed the content to ensure that the wording met standards for patient literacy. The tool was initially developed in English then backward-forward translated into French.

#### Components of the intervention

The cover page of the brochure has an image of a pillbox filled with several medications titled ‘You May Be At Risk’, followed by ‘You are currently taking (name of benzodiazepine)’. Brochures are customized according to each patient’s medication profile. The first page of the intervention lists four true or false questions regarding the safety, side effects, withdrawal symptoms and alternatives to the use of the benzodiazepines, and is entitled ‘Test Your Knowledge’. The second page contains the correct answers as well as an explanation for each statement. The goal is to create cognitive dissonance and challenge the patient’s beliefs for each incorrect answer by incorporating elements of constructivist learning theory into the answers. The third page incorporates a self-assessment component as well as educational facts on potential inappropriate use, side effects, drug-drug interactions and information about physiological changes that occur with age that affect drug metabolism. Suggestions for equally or more effective therapeutic substitutes, as well as evidence-based risks associated with benzodiazepine use in older people, are presented on the fourth and fifth pages. The sixth page highlights one woman’s success story in weaning herself off benzodiazepines. The last page outlines a simple 21-week tapering program that can be adapted to the patient’s medication use. For contrast and visual enhancement, visual such as color shading and several pictures of older adults and medication are used throughout the tool. In order to make sure the intervention is used appropriately, the words ‘Please Consult your Doctor or Pharmacist Before Stopping Any Medication’ appear as a warning in large lettering on four different occasions throughout the tool.

#### Acceptability of the intervention

To determine the readability and comprehension of the information, the tool was field-tested in six focus groups of older adults (n = 60). Based on the focus group feedback, elements of the tool, such as the wording, ordering of the material and the visual presentation were changed in an iterative process until acceptability was reached.

#### Study arms

Participants allocated to the experimental group receive the written educational program via mail immediately following randomization. Telephone follows-ups are conducted 1 week, 1 month, 6 months and 1 year post-intervention, and last 5 to 10 minutes. Participants in the control ‘wait-list’ group are monitored during the first 6 months following randomization and then receive the same intervention as the experimental group.

### Study outcomes

Outcomes are measured at all study follow-up points. At baseline, questionnaires are completed at the participants’ homes during an interview with the research coordinator. Follow-up is by telephone interview with the same coordinator. Self-reported socio-demographic variables, health status variables and prescription details are collected at baseline.

### Primary outcomes

#### Prescription change rate at 6 months

The primary outcome of the study is cessation of benzodiazepines in the 6 months following receipt of the intervention, ascertained by pharmacy renewal profiles and confirmed by patient self-report. A 1-year follow-up will be undertaken to determine whether change rates are sustained over the long-term. The definition of discontinuation will be an absence of any benzodiazepine prescription renewal at the time of the 6-month follow-up. Dose reductions will also be measured and will be defined as ≥50% reductions in the renewal profile for at least 3 consecutive months beginning at the time of the 6-month follow-up. The discontinuation/dose reduction rate among participants in the experimental arm will be compared to the discontinuation/dose reduction rate among participants in the control arm. In this way we will be able to determine the absolute rate of discontinuation attributable to the intervention. This outcome measure pertains to the individual level.

The 6-month period and 1-year follow-up were chosen because although there is no agreement on the time frame of change, the trans-theoretical model supposes that, typically, once people start thinking about changing their behavior, decision and planning of the action is usually done within the following 6 months. Maintenance of the new behavior begins after 6 months of being in the active stage of changing and continues for at least 6 months [59]. Pharmacy profiles, supplied monthly by fax to the research center by the pharmacist, were chosen to measure prescription change rates because of the high amount of information they contain. Pharmaceutical profiles vary in the information they contain between pharmacies of the same chain depending on the owners. However, vital information to determine change rates, such as the date of renewal, the dose and the quantity of the prescription are always listed. Using this objective measure allows comparison and validation of patient reported outcomes, and thus more accurately and objectively determines the effect of the intervention.

### Secondary outcomes

#### Change in risk perception

Change in perception of risk associated with benzodiazepine use will be evaluated through a self-reported measure, along with change in knowledge and change in beliefs. The self-reported measure will consist of participants answering whether they perceived the same, increased or no risk from consumption of their benzodiazepine medication after having read the brochure, and will be collected 1 week post-intervention. Change in knowledge will be measured by comparing the pre- and post-intervention (T1) answers from the four true or false questions in the ‘Test Your Knowledge’ section of the questionnaire. The first statement targets safety of long-term benzodiazepine and reads, ‘(Example: Valium®) … is a mild tranquilizer that is safe when taken for long periods of time’. The second statement focuses on side effects and is phrased, ‘The dose of Valium® that I am taking causes no side effects’. The third statement, focusing on withdrawal, is worded, ‘Without Valium® I will be unable to sleep or will experience unwanted anxiety’, and the fourth, on alternative treatment options, states, ‘Valium® is the best available option to treat my symptoms’. Change in beliefs is measured by comparing the pre- and post-intervention (T1) total scores on the Specific section of the beliefs about medicines questionnaire (BMQ-Specific) adapted for benzodiazepines [60,61]. Statements remained identical to the originals with the exception that the word ‘medicines’ was replaced by ‘benzodiazepine’ in each statement. The beliefs in medications questionnaire is a validated measure used to assess cognitive representations of medications [60,61]. These outcome measures pertain to the individual level.

Change in risk perception was chosen as a secondary outcome in order to reflect the behavior motivation hypothesis described earlier. As patient reported outcomes are not always objective, two additional and more objective outcomes were chosen to evaluate risk perception: change in knowledge and change in beliefs about benzodiazepines. This was done because a common idea in models of risk perception states that risk is perceived from two dimensions: knowledge of and beliefs about that risk, as mentioned earlier. The rationale for choosing the score for the knowledge questionnaire was that it allows a quantification of the knowledge transfer aspects of the intervention. The rationale for choosing the BMQ-Specific instrument to measure beliefs relates to its ability to isolate and score participants’ beliefs about a specific medication; both in terms of the dangers and concerns participants have regarding their prescription (Specific-Concerns), and the necessity they attribute to this same prescription (Specific-Necessity). The BMQ-Specific consists of two 5-item factors belonging to each sub-score. Participants indicate their degree of agreement with each statement on a 5-point Likert scale (1 = strongly disagree to 5 = strongly agree). Both scales are then summed into their respective scores (5 to 25 scale) with higher scores indicating stronger beliefs in that concept. A necessity-concerns differential can also be derived from these scales by subtracting the concern sub-score from the necessity sub-score. This differential can be considered as the cost benefit analysis for each patient, where costs (concerns) are weighed against perceived benefits (necessity beliefs) [60,61].

#### Change in self-efficacy

The second secondary outcome measure will be change in self-efficacy. Self-efficacy will be measured pre- and post-intervention (T1) with the medication reduction self-efficacy scale, a scale that was developed and tested in the context of previous benzodiazepine tapering studies [62]. Participants will indicate their level of confidence for achieving a pre-determined medication reduction goal on a scale of 0 to 100 (0 = not at all confident to 100 = extremely confident), which is based on Bandura’s original guidelines for the development of task-specific self-efficacy scales. Post-intervention, participants will also be asked to rate on this same scale their level of confidence about eventually discontinuing using the tapering program provided. This outcome measure pertains to the individual level. The rationale is that self-efficacy gives a clear indication of a patient’s belief about their capability to discontinue benzodiazepines and may be a potential predictor of benzodiazepine discontinuation.

#### Initiation of discussion with a physician or pharmacist about the decision to taper benzodiazepines

The third secondary outcome will be the potential of the intervention to activate participants to discuss safer prescribing options with their physician or pharmacist. At T1 to T3 participants will be asked to indicate: if they had spoken to friends and/or family about the intervention, and if they had spoken to or intended to discuss medication discontinuation with either their physician or pharmacist. Reactions and results of these behaviors will be noted. These intentions are considered as measures of self-initiated medication risk reduction behaviors. This outcome measure pertains to the individual level.

The intervention was designed to target consumers directly as catalysts for engaging physicians and pharmacists in collaborative discontinuation of their benzodiazepine drugs or other inappropriate medications. Observing this outcome will allow us to determine the intervention’s potential for engaging participants in collaborative medication management. Furthermore, it will also allow us to identify at which point the intervention failed, and whether psychological dependence on the part of consumers or obstructive behavior on the part of the physicians or pharmacists was the cause of the intervention’s failure.

### Sample size

The main question driving the sample size for this study is whether chronic inappropriate medication users who receive the knowledge transfer tool are more likely to discontinue use at 6-month follow-up compared to users who do not receive the intervention. A systematic review was undertaken to identify similar studies and compare discontinuation rates for benzodiazepine drugs. Inclusion criteria were: rigorous randomized controlled trial methodology, inclusion of adults aged 65 years and older, community setting, a non-imposed intervention, and interventions that targeted inappropriate benzodiazepine prescriptions and included a prescription discontinuation measure. Eight studies met the inclusion criteria and were used in the sample size calculation estimates. Many other studies were identified that presented very different estimates, however these varied greatly in setting, population or measure and were irrelevant to the current study.

We expect our intervention to achieve a rate of discontinuation that is at least as great as that achieved in previous studies by medication review by pharmacist and contact with physician (range 19 to 24%, mean 22%) [29,43,63] or by simple discontinuation letters (range 13 to 20%, mean 16%) [47,64-67]. However, it is possible that individuals who do not receive the intervention may have rates of discontinuation as high as 6% for inappropriate prescriptions (range 2 to 6%, mean 4%) [29,43,47,64-66]. Our study will therefore be powered to detect a minimal 20% increase in inappropriate medication discontinuation due to use of the intervention and an absolute minimal rate of discontinuation of 25%. Based on an alpha of 0.05 and 80% power to detect a 20% difference, 58 participants are needed for each group. To detect greater differences, a lower sample size is needed. However, due to the cluster design of this study, adjustments need to be made to account for both clustering and for the effect of the coefficient of variation of the cluster size [68]. Based on current recruitment data (16 clusters, cluster sizes 6 to 27), the coefficient of variation was established at 0.527 using the minimum/maximum cluster size estimation method [68] and estimated intra-cluster correlation set at 0.05. After computing the coefficient by which to multiply our sample size to account for these factors we obtained 1.79 [69]. Current loss to follow-up in the study (in the first 185 recruited participants) was established at 9%. Therefore 114.2 (58 × 1.79 × 1.10 = 114.2) participants will be needed for each group. A sample of 250 individuals will be recruited.

### Analysis plan

Data will be analyzed using an intention-to-treat approach. Descriptive statistics (means, proportions) will first be calculated to assess the balance between the groups on important confounders, such as age, sex, health status, baseline beliefs about medications and benzodiazepine use. In order to answer the main research question driving this study - whether an educational intervention targeting consumers directly as catalysts for engaging physicians and pharmacists in collaborative discontinuation achieves an inappropriate prescription discontinuation rate of at least 20% compared to usual care - we will use a marginal model estimated via generalized estimating equations (GEE) with a binary outcome and an identity link, with an exchangeable correlation structure to account for correlation between participants in the same cluster [69]. Risk differences between the control and experimental groups will be calculated and the robust variance estimator will be used to estimate the associated 95% confidence interval (CI) and *P* value [70]. As a sensitivity analysis (and/or if any confounders are unbalanced between the groups), we will estimate the risk difference for the intervention via a marginal model estimated via GEE with an exchangeable correlation structure. The robust variance estimator will again be used. In secondary analyses, we will calculate risk differences in subgroups of interest (for example, very older people, women, baseline beliefs about medication and degree of polypharmacy). The analysis will be carried out at both the cluster and individual levels.

In order to determine whether the patient intervention altered beliefs about the necessity-concern ratio, knowledge or risk perception for the inappropriate prescriptions, as well as self-efficacy, paired t-tests will be used to evaluate change scores pre- and post-intervention. The potential of the intervention to engage participants in preventive health behaviors will be evaluated via chi-square tests comparing intervention and control groups. These analyses will be carried out at the individual level.

## Discussion

To date there is no effective or sustainable approach to reduce benzodiazepine use in older adults [24-42]. Previous research on strategies to reduce benzodiazepine consumption has applied paternalistic approaches to patient care, similar to the ‘top-down’ managerial approach described in management and organizational development theory [71,72]. An example of this approach is when physicians acquire warning letters from study investigators and send these letters to patients asking them to schedule an office visit to discuss benzodiazepine discontinuation. Our educational intervention draws on theories of self-management and collaborative doctor-patient partnerships, and provides a means to test a ‘bottom-up’ change strategy [71,72]. In the bottom-up model, patients drive prescription decisions from information gathered on the Internet, through friends or via an accredited academic body. To our knowledge, no published study to date has targeted the patient as a driver of safer prescribing practices. By directly empowering chronic users with knowledge about risks, suggestions for lower-risk therapeutic options and self-efficacy for implementing tapering protocols, we hope to imitate the success of individually targeted anti-smoking campaigns [73].

To maintain the generalizability of the findings from our study, exclusion criteria have been kept to a minimum. In order to fulfill recruitment needs, no limits on cluster size were imposed to pharmacies meeting the cluster eligibility criteria. Since some pharmacies identified over 200 potential participants, while others barely covered the 50 potential candidate minimum to qualify as a cluster, cluster sizes are expected to vary. However, this was considered both in the sample size calculations and analyses.

The study has been designed as a pragmatic trial that takes place in the real-world setting. The intervention is theoretically-based and incorporates a practical and contemporary learning and psychological approach to help participants overcome hard-to-achieve lifestyle modifications. Thus, we expect that implementing an educational intervention trial in a practical setting will yield both internally and externally valid evidence for reducing inappropriate benzodiazepine use, by directly targeting and activating community-dwelling older adults in a previously unexplored approach.

### Trial status

The trial is currently recruiting participants and was approximately 80% complete at time of publication.

## Abbreviations

BMQ-Specific: Beliefs about Medicines Questionnaire - Specific segment; CI: Confidence interval; GAI: Geriatric Anxiety Inventory; GEE: Generalized estimating equations; GHS: General health status; MoCA: Montreal Cognitive Assessment; PHQ: Patient Health Questionnaire; SMAF: Functional autonomy measurement system.

## Competing interests

The authors declare that they have no competing interests.

## Authors’ contributions

CT conceived the study, participated in its design and coordination, and helped to revise the manuscript. PM was involved in drafting and revising the manuscript. RT and SA contributed to the conception and design of the study, and were involved in manuscript revision. All authors read and approved the final manuscript.
